# Use of retinal ischemic perivascular lesions (RIPLS) as a biomarker for cardiovascular disease – a systematic review and meta-analysis

**DOI:** 10.1186/s40942-025-00782-2

**Published:** 2025-12-24

**Authors:** Fatima Zahra, Manahil Malik, Khadijah Abid, Karim F. Damji, Haroon Tayyab

**Affiliations:** 1 Department of Ophthalmology and Visual Sciences, Aga Khan University, Stadium Road, Karachi, 74800 Pakistan; 2 Department of Ophthalmology and Visual Sciences, University of Alberta, Edmonton, Canada

**Keywords:** Retinal ischemic perivascular lesions, Cardiovascular disease, Optical coherence tomography, Retinal imaging

## Abstract

**Background:**

This study aims to evaluate the association between retinal ischemic perivascular lesions (RIPLs) detected by optical coherence tomography (OCT) and the risk of all-cause cardiovascular morbidity.

**Methods:**

A systematic review and meta-analysis were performed. PubMed MEDLINE, Scopus, and Cochrane CENTRAL were searched for observational studies from January 2015 to March 2025 assessing RIPLs and cardiovascular disease (CVD). Odds ratios (ORs) with 95% confidence intervals (CIs) were pooled using a random-effects model.

**Results:**

Of 61 studies screened, six met inclusion criteria and four were included in the meta-analysis (total *n* = 710). The pooled OR for CVD morbidity associated with RIPLs was 2.8 (95% CI: 1.98–3.95), indicating over a twofold increased risk. Heterogeneity was minimal (I² = 0%).

**Conclusions:**

RIPLs detected by OCT are significantly associated with increased cardiovascular risk. Due to OCT’s non-invasive and accessible nature, RIPLs may be a useful screening biomarker for early detection of CVD.

**Supplementary Information:**

The online version contains supplementary material available at 10.1186/s40942-025-00782-2.

## Introduction

Cardiovascular disease (CVD) remains the leading cause of mortality and morbidity around the world, accounting for millions of deaths annually and significantly impacting quality of life [1]. As per current statistics, approximately 9% of individuals over the age of 20 are affected by CVD highlighting the critical need for effective early identification and intervention strategies. While lifestyle modifications and pharmacological treatments can effectively reduce the risk of cardiovascular events, many patients remain undiagnosed until they experience severe adverse events such as myocardial infarction or stroke [1–3]. This underscores the importance of detecting biomarkers of incipient ischemia that can facilitate timely diagnosis and management of cardiovascular diseases.

The middle retinal layer, which is the neurosensory retinal layer, is very sensitive to any changes in blood flow. It consists of Inner Nuclear Layer (INL) and Outer Plexiform Layer (OPL). Blood supply in this region is through a network of intermediate and deep capillary plexus. Hypoperfusion due to any reason such as decreased ejection fraction, carotid stenosis or low blood flow to the capillary plexus may lead to retinal layer ischemia. Acute ischemia is seen as transient lesions termed as Paracentral Acute Middle Maculopathy (PAMMs) [4].

Chronic ischemia leads to atrophy of middle retinal layer and development of permanent lesions termed as Retinal Ischemic Perivascular Lesions (RIPLs). RIPLs are seen are seen as focal INL thinning, OPL displacement and Outer Nuclear Layer (ONL) expansion on optical coherence tomography (OCT) [4, 5].

When discussing RIPLs, PAMM is another comparable ischemic retinal pathology. PAMM appear as hyperreflective bands in INL on OCT. While RIPLs are irreversible, chronic and asymptomatic lesions that make them more reliable for OCT imaging, PAMM are revisable lesions reflecting ischemia in deep capillary plexus (DCP), with scotoma as a clinical presentation. Their transient nature (disappears over weeks) makes them unreliable for being a permanent biomarker detected through OCT [6–9]. With continuing advances in the OCT resolution, these biomarkers will be more readily visible for detection and likely to enhance training of artificial intelligence (AI) models.

Emerging evidence depicts a significant correlation between cardiovascular disease and the occurrence of retinal vascular occlusions [10–12]. The retina, characterized by its multi-layered formation and intricate capillary and vascular network, serves as a potential indicator of systemic vascular health [13]. Specifically, retinal ischemic perivascular lesions (RIPLs) have emerged as promising biomarker of underlying subclinical cardiovascular pathology. Noninvasive imaging modalities, like spectral domain optical coherence tomography (SD-OCT), allow for the detailed visualization of these lesions at a resolution of 5 to 7 microns, enabling the detection of retinal abnormalities that may signal ischemic events [14]. In future, high-resolution OCT may add even better visualization of subtle ischemic retinal changes.

In 2021, Long and co-authors coined the term ‘RIPL’ based on SD-OCT imaging characteristics. (Supplementary Material 1) [13–15]. These lesions have been recorded in various conditions associated with cardiovascular risk factors, including retinal artery and vein occlusions, hypertension, and other ischemic conditions. Furthermore, advanced imaging techniques like OCT angiography have uncovered blood flow signal voids that support the ischemic nature of these lesions. This reflects localized retinal ischemia but could also serve as markers of systemic cardiovascular disease [16–18]. 

This systematic review and meta-analysis aim to explore the role of RIPLs in detecting cardiovascular ischemia and to assess its reliability as a biomarker for CVD. To our knowledge, no such meta-analysis exists and our study aims to address this important gap in literature. We will summarize and synthesize findings from existing literature to evaluate the strength of evidence supporting this association.

## Methods

### Data source and search strategy

This study was conducted in accordance with the preferred reporting items for systematic review and meta-analysis (PRISMA) guidelines. It is a prognostic systematic review, which aims to evaluate RIPLs as a biomarker for detection of CVD.

PubMed, MEDLINE, Scopus and Cochrane CENTRAL electronic databases were searched from January 2015 till March 2025, using the search string attached in Supplementary Material 2.

The search window (2015–2025) was selected because RIPLs were first described as a biomarker in 2021, and meaningful OCT-based descriptions only emerged in the past decade. While this time restriction improves relevance, it may introduce bias by excluding older imaging studies.

Additionally, we manually screened reference list of relevant articles including review articles and observational studies to find additional suitable studies.

The review followed a predefined PECO framework, which helped develop the search terms and the overall search strategy:

- Population: Adults undergoing OCT imaging.
- Exposure: Presence of RIPLs.
- Comparator: Absence of RIPLs.
- Outcome: Cardiovascular diseases (coronary artery disease, myocardial infarction, atrial fibrillation, carotid stenosis, peripheral arterial disease, acute coronary syndromes).

### Study selection

The following inclusion criteria were used:

- Studies investigating RIPLs as a biomarker for cardiovascular disease detected via OCT.
- Cardiovascular diseases include the following: Coronary heart disease, peripheral arterial disease, atherosclerosis, acute coronary artery syndrome, heart arrhythmias –such as atrial fibrillation.
- Observational, cross-sectional, case series.
- Studies published from January 2015- March 2025 in English, conducted globally.

Exclusion criteria:

- Studies without control group were not included for meta-analysis.

### Data extraction and assessment of study quality

The articles retrieved from systematic search were exported to PRISMA software where duplicates were removed. The remaining articles were meticulously screened by two independent reviewers (FZ and MM) and articles that met the inclusion criteria were selected. Initial short-listing was done based on title and abstract screening, which was then followed by full text review. A third reviewer (HT) was consulted in case there were any discrepancies. The following outcomes were extracted: Coronary heart disease, Peripheral arterial disease, Heart arrhythmias –such as atrial fibrillation, Atherosclerosis, Stroke and Transient Ischemic Attack (TIA), Acute Coronary Artery Syndrome. The Newcastle-Ottawa scale was used to assess the quality of observational studies.

Major confounders including age, hypertension, diabetes mellitus, dyslipidemia, chronic kidney disease, and smoking status were prespecified during protocol development as clinically relevant variables potentially influencing the association between RIPLs and cardiovascular disease. These variables were extracted when reported; however, inconsistent reporting across studies limited the feasibility of formal subgroup or heterogeneity analyses.

OCT modality, device manufacturer, scan protocol, and RIPL grading methodology were extracted for all studies. All included studies used spectral-domain OCT and applied similar macular scanning protocols with comparable definitions of RIPLs. Intergrader agreement was recorded when available and ranged from moderate to high across studies. Only studies employing masked grading were included.

### Statistical analysis

RevMan (Version 5.4. Copenhagen: The Nordic Cochrane Centre, The Cochrane Collaboration, 2020) was used for conducting the meta-analysis. Results from observational studies were presented as odds ratio (OR) with 95% confidence intervals (CIs) and were pooled using random effect model. Forest plot was generated to visualize the results of pooling. Heterogeneity in meta-analysis was evaluated using Higgins I^2^. A value of less than 50% was accepted. A P-value of less than 0.05 was considered significant for all results.

## Results

### Literature search results

Initial results of three electronic databases resulted in 61 potential studies. After exclusion, six observational studies were initially finalized for analysis and two studies were further excluded from meta-analysis due to missing a control group. A total of 4 studies (Supplementary Material 3) were therefore included in the final meta-analysis. The PRISMA flow chart (Supplementary Material 4) summarizes the result of our literature search.

### Study characteristics and quality assessment

Combing these studies resulted in a total of 710 patients which were included in the meta-analysis. Baseline characteristics of these studies are summarized in Supplementary Material 5.

Detailed quality assessment of each study is given in Supplementary Material 6. This systematic review has been registered with PROSPERO (Reference ID CRD420250608019).

### All-cause morbidity

A total of 4 studies were included in the meta-analysis evaluating association between RIPLs and all cause CVD morbidity.

The pooled odds ratio (OR), calculated using random-effects model, was 2.8 (95% CI:1.98–3.95). This indicates that individuals with RIPLs have more than twofold increased risk of all-cause CVD morbidity compared to those without RIPLs. Forest plot provided in Supplementary Material 7. The forest plot displays a consistent direction of effect across all studies, with each study showing a positive association between RIPLs and CVD. No significant outliers were observed.

The lack of heterogeneity (I^2^ = 0%) signifies that there is minimal variation in the effect size of all studies. This supports the consistency of pooled estimate and strengthens the validity of our findings. The p value of < 0.05 indicates that the results are significant.

These findings support RIPLs as a potential non-invasive biomarker associated with CVD.

## Discussion

Our study statistically demonstrates that RIPLs are more frequently observed in individuals with CVD. The study by Bousquet et al. [4] contributed the highest weight (58.2%) in the meta-analysis. This is likely due to its large sample size of 317 patients and the narrow confidence interval (1.91–4.71), indicating a high level of precision in its effect estimate. In contrast to this, Drakopoulos et al. [6] contributed the lowest weight (3.4%), with the widest confidence interval (0.62–25.81), which was not statistically significant. This low weight is most likely due to the study’s small sample size of only 36 patients (14 controls and 22 cases). This resulted in a less precise and more variable estimate. However, when the studies were combined in the meta-analysis, the pooled estimate reached statistical significance, showing the importance of combining data, even when individual studies may be underpowered.

The study by Long et al. [5] reported the highest odds ratio (5.34), suggesting the strongest association between RIPLs and CVD among the included studies. An interesting feature of this study is its classification of RIPLs into three categories based on lesion count (1 lesion, 2 lesions, and ≥ 3 lesions). For the purposes of this meta-analysis, we used the odds ratio corresponding to the group with three or more lesions, which had the highest specificity of 93.4%. To further explore this association, we conducted two separate meta-analysis with OR of 1 lesion versus 2 lesions however the final results remained consistent and there was no significant difference. This may suggest that number of lesions is not the primary and only determinant, however further studies are needed to explore this association.

In contrast to the findings of Long et al., Bakhoum et al. [7] reported the lowest odds ratio (OR = 1.91) in the included studies; however, the association remains in the same direction, supporting the overall trend. Bakhoum et al.‘s study contributes the second highest weight (29.2%) to the meta-analysis, showing its stronger impact on the final results.

A narrative review by Limoli et al. includes several of the studies mentioned above and arrives at a conclusion which correlates with our findings, supporting use of RIPLs as an indicator of underlying CVD [8]. 

### Synthesis of studies excluded from meta-analysis

Wang et al. conducted a study with 474 patients, indicating that an increased number of RIPLs was associated with a greater degree of carotid artery stenosis. However, this study could not be included into our meta-analysis as there was no control group or odds ratio, both of which are required for meta-analysis [19]. 

Kwapong et al. in their study, explored the correlation between RIPLs and single subcortical cerebral infarcts. While their findings contribute to the broader understanding of RIPLs, the study was not included in our meta-analysis, as its primary focus was on neurological rather than cardiovascular outcomes [20]. 

Two more studies by, Madala et al. [21] and Yeo et al. [22] could not be included in our meta-analysis as they reported as case series. However, both studies highlight a potential association between RIPLs and CVD, which further supports the overall direction of our findings.

A closer look at the data revealed, eight out of eleven hypertensive patients included in Madala et al. [21] with presence of RIPLs, had a range of underlying CVD. Of these eight patients, three underwent medical therapy, while two had surgical interventions, including coronary artery bypass grafting (CABG) and carotid artery stent placement. These clinical observations suggest that incidental detection of RIPLs may prompt further systemic evaluation.

In the study by Yeo et al. [22], out of 11 patients with incidentally identified RIPLs, 10 (90.9%) had either diagnosed CVD or significant cardiovascular risk factors including; type 2 diabetes mellitus, hypertension, CAD, arrhythmia, cerebrovascular events, CAS, and peripheral vascular disease. These findings support the use of RIPLs as an indicator of possible underlying CVD.

The study populations presents a range of racial and ethnic groups (White, African American, Asian, Hispanic) [5, 7, 22] adding to the generalizability of findings. Sample sizes varied from small case series (*n* = 11) to large cohorts (*n* = 317), with average ages mostly in the late 60s to early 70s. Although gender distribution varied, females were generally underrepresented. Despite heterogeneity in study design and outcome measures, the presence of RIPLs, was a consistent finding in patients at risk of CVD.

Across the included studies, racial and ethnic representation was broadly similar, with White individuals forming the largest proportion, followed by Asian and Hispanic populations, while African American representation was comparatively low. None of the studies reported differential RIPL prevalence or outcomes by race or ethnicity.

Female participants were consistently underrepresented across studies. This pattern likely reflects the underlying epidemiology of cardiovascular disease, which is more prevalent in males in the age range of the included cohorts (predominantly late 60s to early 70s). Conditions such as coronary artery disease, atrial fibrillation, and carotid artery stenosis frequent diagnoses among the study populations are also historically more common in men, which may account for their higher representation.

RIPLs may help identify individuals who warrant further cardiovascular evaluation. It can help in early identification of individuals at risk of CVD. An interdisciplinary collaboration between ophthalmologist, cardiologists and neurologists can help prevent life-threatening cardiovascular and stroke events and facilitate in improving patient outcome. Since it is non-invasive, convenient to use and widely available, healthcare workers can be trained to use it for screening in community settings specially in low-and middle-income countries (LMICs) and regions with limited access to physicians.

Integration of Artificial Intelligence (AI) can enhance the usability of this biomarker. AI based algorithms can be used as standardized screening tool, that can accurately detect the size, number and morphology of RIPLs on OCT and may assist in standardizing detection of RIPLs and facilitate timely referral for systemic assessment. It can also be used in remote areas for CVD screening and help reduce clinical workload.

This study consists of several strengths that distinguish it from previous work. To our knowledge this is the first meta-analysis compiling the results of available literature on RIPLs and its association with CVD, statistically evaluating the conclusion. Our study has zero heterogeneity among the included studies, which indicates a high degree of consistency in the findings. Additionally, no outliers were identified, and all studies demonstrated effect estimates in the same direction, which strengthens the reliability and validity of the meta-analytic results.

The study includes a diverse population representing White, Hispanics, Black and Asian ethnicities. This adds to the generalizability of the results.

The study was conducted using the PRISMA guidelines which ensures the reliability and high quality of the research that has been conducted.

However, this study is subject to certain limitations. Firstly, it has a small sample size for meta-analysis. Several potentially relevant studies were excluded from meta-analysis due to the absence of a control group. These studies are mentioned in the discussion section.

The literature search was limited to four major databases i.e. PUBMED, Scopus, MEDLINE, and the Cochrane Library to ensure the inclusion of high-quality studies. However, this may have led to the exclusion of relevant studies indexed elsewhere.

Additionally, a publication bias may exist; however, due to the small number of studies (*n* < 10), a funnel plot could not be generated to formally assess this bias.

A lack of standardized methods for detecting RIPLs via OCT might have introduced variability in the reported findings. Moreover, while all included studies explored the impact of CVD on RIPLs, the specific types of CVD varied, ranging from atrial fibrillation to myocardial infarction. Due to the limited number of studies, a subgroup analysis for each cardiovascular condition could not be performed and therefore we had to combine outcomes into a single endpoint.

Importantly, although the associations between RIPLs and CVD were consistent, the evidence does not permit causal or prognostic inference. The cross-sectional study designs limit our ability to determine temporal relationships between RIPLs and cardiovascular pathology. Therefore, RIPLs should currently be viewed as a potential adjunctive biomarker that may prompt further evaluation rather than as an established predictor of cardiovascular risk.

## Conclusion

The findings of this review demonstrate that RIPLs detected on OCT are associated with CVD across several independent studies. While these associations are consistent and biologically plausible, the evidence is derived from cross-sectional and retrospective observational studies and therefore cannot support causal or prognostic conclusions. RIPLs may represent a useful adjunctive biomarker that raises suspicion for underlying cardiovascular disease, but prospective studies are needed to determine their predictive value and clinical utility.

## Supplementary Information

Below is the link to the electronic supplementary material.

Supplementary Material 1

Supplementary Material 2

Supplementary Material 3

Supplementary Material 4

Supplementary Material 5

Supplementary Material 6

Supplementary Material 7

## Data Availability

No datasets were generated or analysed during the current study.
