# Supplementary material for: Use of retinal ischemic perivascular lesions (RIPLS) as a biomarker for cardiovascular disease – a systematic review and meta-analysis
Source: Int J Retina Vitreous. 2025 Dec 24;12:15. doi: 10.1186/s40942-025-00782-2 (PMC12837118; doi:10.1186/s40942-025-00782-2)

**Supplementary Material 1: Figure 1**

**Article Title:**
Use of Retinal Ischemic Perivascular Lesions (RIPLs) as a Biomarker for Cardiovascular Disease – A Systematic Review and Meta-analysis

**Journal:**
International Journal of Retina and Vitreous

**Authors:**
Fatima Zahra, Manahil Malik, Khadijah Abid, Karim F. Damji, Haroon Tayyab

**Corresponding Author:**
Dr. Haroon Tayyab

**Affiliation:**
Department of Ophthalmology, Aga Khan University, Karachi, Pakistan

**E-mail Address:**
haroon.tayyab@aku.edu

Figure 1: Spectral-domain optical coherence tomography (SD-OCT) B-scan of a normal eye (top) compared with OCT B-scans from patients with retinal ischemic perivascular lesions (RIPLs) (bottom).
 The top panel shows a normal B-scan illustrating the retinal layers. The bottom panel displays three independent cases of RIPLs, marked by focal thinning of the inner nuclear layer (INL) and expansion of hypo reflectivity in the outer nuclear layer (ONL). Adapted from Bousqet et al.[13]


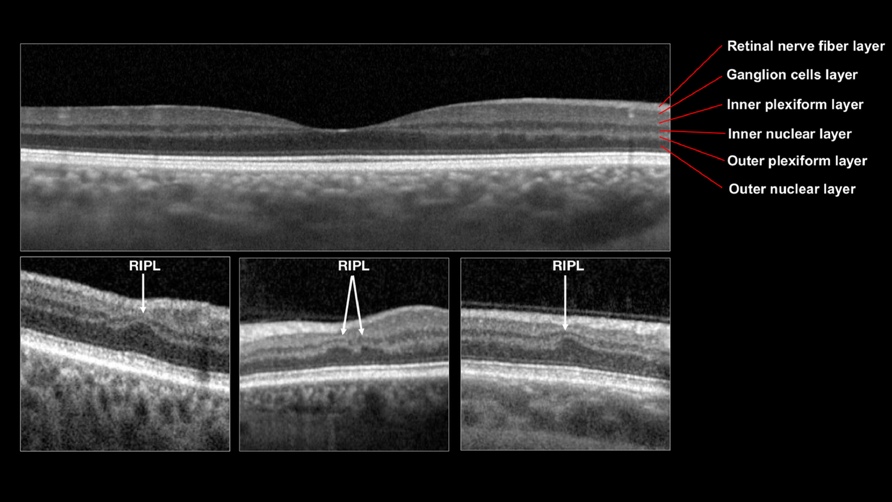

Supplement: Supplementary file 1 — Supplementary Material 1 [file 40942_2025_782_MOESM1_ESM.docx]
