# Supplementary material for: Use of retinal ischemic perivascular lesions (RIPLS) as a biomarker for cardiovascular disease – a systematic review and meta-analysis
Source: Int J Retina Vitreous. 2025 Dec 24;12:15. doi: 10.1186/s40942-025-00782-2 (PMC12837118; doi:10.1186/s40942-025-00782-2)
Supplement: Supplementary file 2 — Supplementary Material 2 [file 40942_2025_782_MOESM2_ESM.docx]

**Supplementary Material 2: Search String**

**Article Title:**
Use of Retinal Ischemic Perivascular Lesions (RIPLs) as a Biomarker for Cardiovascular Disease – A Systematic Review and Meta-analysis

**Journal:**
International Journal of Retina and Vitreous

**Authors:**
Fatima Zahra, Manahil Malik, Khadijah Abid, Karim F. Damji, Haroon Tayyab

**Corresponding Author:**
Dr. Haroon Tayyab

**Affiliation:**
Department of Ophthalmology, Aga Khan University, Karachi, Pakistan

**E-mail Address:**
haroon.tayyab@aku.edu

Supplementary Material 2: Search String

("retinal ischemic perivascular lesion"[All Fields] OR (("retinaldehyde"[Supplementary Concept] OR "retinaldehyde"[All Fields] OR "retinal"[All Fields] OR "retinaldehyde"[MeSH Terms] OR "retina"[MeSH Terms] OR "retina"[All Fields] OR "retinally"[All Fields] OR "retinals"[All Fields] OR "retinitis"[MeSH Terms] OR "retinitis"[All Fields]) AND "perivascular"[All Fields] AND ("ischaemia"[All Fields] OR "ischemia"[MeSH Terms] OR "ischemia"[All Fields] OR "ischaemias"[All Fields] OR "ischemias"[All Fields])) OR "retinal ischemia"[All Fields] OR "retinal ischemic lesions"[All Fields] OR "retinal vascular lesions"[All Fields] OR "RIPL"[All Fields] OR (("retinaldehyde"[Supplementary Concept] OR "retinaldehyde"[All Fields] OR "retinal"[All Fields] OR "retinaldehyde"[MeSH Terms] OR "retina"[MeSH Terms] OR "retina"[All Fields] OR "retinally"[All Fields] OR "retinals"[All Fields] OR "retinitis"[MeSH Terms] OR "retinitis"[All Fields]) AND ("microinfarct"[All Fields] OR "microinfarction"[All Fields] OR "microinfarctions"[All Fields] OR "microinfarcts"[All Fields])) OR "retinal infarct*"[All Fields] OR "retinal ischemic event*"[All Fields] OR ("inner"[All Fields] AND ("nuclear"[All Fields] OR "nuclears"[All Fields]) AND ("layer"[All Fields] OR "layer s"[All Fields] OR "layered"[All Fields] OR "layering"[All Fields] OR "layerings"[All Fields] OR "layers"[All Fields]) AND "infarct*"[All Fields]) OR "retinal capillary ischemia"[All Fields] OR "paracentral acute middle maculopathy"[All Fields] OR "PAMM"[All Fields]) AND ("cardiovascular disease*"[All Fields] OR "cardiovascular event*"[All Fields] OR "cardiovascular risk factor*"[All Fields] OR "ischemic heart disease"[All Fields] OR "coronary artery disease"[All Fields] OR "coronary heart disease"[All Fields] OR "heart disease*"[All Fields] OR "myocardial infarction"[All Fields] OR "hypertension"[All Fields] OR "high blood pressure"[All Fields] OR "atherosclerosis"[All Fields] OR "vascular disease*"[All Fields] OR "carotid artery stenosis"[All Fields] OR "carotid plaque*"[All Fields] OR "multivessel coronary artery disease"[All Fields] OR "subclinical cardiovascular disease"[All Fields] OR "cardiac disease*"[All Fields]) AND ("detection"[All Fields] OR "screening"[All Fields] OR "diagnosis"[All Fields] OR "early detection"[All Fields] OR "biomarker*"[All Fields] OR "cardiovascular screening"[All Fields] OR (("cardiacs"[All Fields] OR "heart"[MeSH Terms] OR "heart"[All Fields] OR "cardiac"[All Fields]) AND ("risk"[MeSH Terms] OR "risk"[All Fields]) AND ("detect"[All Fields] OR "detectabilities"[All Fields] OR "detectability"[All Fields] OR "detectable"[All Fields] OR "detectables"[All Fields] OR "detectably"[All Fields] OR "detected"[All Fields] OR "detectible"[All Fields] OR "detecting"[All Fields] OR "detection"[All Fields] OR "detections"[All Fields] OR "detects"[All Fields])) OR "retinal biomarker*"[All Fields] OR "predictor*"[All Fields] OR "clinical marker*"[All Fields] OR "cardiovascular marker*"[All Fields]) AND ("spectral domain optical coherence tomography"[All Fields] OR "SD-OCT"[All Fields] OR "optical coherence tomography"[All Fields] OR "OCT"[All Fields] OR "retinal imaging"[All Fields] OR "noninvasive retinal imaging"[All Fields] OR "OCT angiography"[All Fields] OR "OCTA"[All Fields] OR "fluorescein angiography"[All Fields] OR "FA"[All Fields] OR "fundus photography"[All Fields] OR "high-resolution imaging"[All Fields] OR "imaging modality"[All Fields])
