# Supplementary material for: Use of retinal ischemic perivascular lesions (RIPLS) as a biomarker for cardiovascular disease – a systematic review and meta-analysis
Source: Int J Retina Vitreous. 2025 Dec 24;12:15. doi: 10.1186/s40942-025-00782-2 (PMC12837118; doi:10.1186/s40942-025-00782-2)
Supplement: Supplementary file 3 — Supplementary Material 3 [file 40942_2025_782_MOESM3_ESM.docx]

**Supplementary Material 3 : Articles References**

**Article Title:**
Use of Retinal Ischemic Perivascular Lesions (RIPLs) as a Biomarker for Cardiovascular Disease – A Systematic Review and Meta-analysis

**Journal:**
International Journal of Retina and Vitreous

**Authors:**
Fatima Zahra, Manahil Malik, Khadijah Abid, Karim F. Damji, Haroon Tayyab

**Corresponding Author:**
Dr. Haroon Tayyab

**Affiliation:**
Department of Ophthalmology, Aga Khan University, Karachi, Pakistan

**E-mail Address:**
haroon.tayyab@aku.edu

**Supplementary Information (SI) 2: Studies included in Meta Analysis**

Study Author

1. Long et all 2021^1^
2. Drakopoulos et al 2023 ^2^
3. Bakhoum et al 2023^3^
4. Bousquet et al 2024^4^

References

1. Long CP, Chan AX, Bakhoum CY, et al. Prevalence of subclinical retinal ischemia in patients with cardiovascular disease – a hypothesis driven study. *eClinicalMedicine*. 2021;33. doi:10.1016/j.eclinm.2021.100775

2. Drakopoulos M, Zhang DL, Cheng BT, et al. Swept-source optical coherence tomography angiography metrics of retinal ischaemic perivascular lesions in patients being evaluated for carotid artery stenosis and controls. *BMJ Open Ophthalmol*. 2023;8(1):e001226. doi:10.1136/bmjophth-2022-001226

3. Bakhoum CY, Madala S, Lando L, et al. Retinal Ischemic Perivascular Lesions in Individuals With Atrial Fibrillation. *J Am Heart Assoc*. 2023;12(16):e028853. doi:10.1161/JAHA.122.028853

4. Bousquet E, Santina A, Au A, et al. Retinal Ischemic Perivascular Lesions Are Associated With Myocardial Infarction in Patients With Coronary Artery Disease. *Am J Ophthalmol*. 2024;264:224-228. doi:10.1016/j.ajo.2024.03.017
