# Supplementary material for: Use of retinal ischemic perivascular lesions (RIPLS) as a biomarker for cardiovascular disease – a systematic review and meta-analysis
Source: Int J Retina Vitreous. 2025 Dec 24;12:15. doi: 10.1186/s40942-025-00782-2 (PMC12837118; doi:10.1186/s40942-025-00782-2)
Supplement: Supplementary file 4 — Supplementary Material 4 [file 40942_2025_782_MOESM4_ESM.docx]

**Supplementary Material 4: PRISMA FLOW DIAGRAM**

**Article Title:**
Use of Retinal Ischemic Perivascular Lesions (RIPLs) as a Biomarker for Cardiovascular Disease – A Systematic Review and Meta-analysis

**Journal:**
International Journal of Retina and Vitreous

**Authors:**
Fatima Zahra, Manahil Malik, Khadijah Abid, Karim F. Damji, Haroon Tayyab

**Corresponding Author:**
Dr. Haroon Tayyab

**Affiliation:**
Department of Ophthalmology, Aga Khan University, Karachi, Pakistan

**E-mail Address:**
haroon.tayyab@aku.edu

**Identification of studies via databases and registers**

Records removed *before screening*:

Duplicate records removed (n = 0 )

Records marked as ineligible by automation tools (n = 0 )

Records removed for other reasons (n = 0 )

Records identified from*:

Databases (n = 61)

**Identification**

Records screened

(n = 61)

Records excluded

(n = 53 )

Reports sought for retrieval

(n = 8)

Reports not retrieved

(n = 0 )

**Screening**

Reports assessed for eligibility

(n = 8)

Reports excluded:

CVD not primary outcome(n=2)

Studies included in review

(n =6)

**Included**

PRISMA flow diagram
