# Supplementary material for: Use of retinal ischemic perivascular lesions (RIPLS) as a biomarker for cardiovascular disease – a systematic review and meta-analysis
Source: Int J Retina Vitreous. 2025 Dec 24;12:15. doi: 10.1186/s40942-025-00782-2 (PMC12837118; doi:10.1186/s40942-025-00782-2)
Supplement: Supplementary file 5 — Supplementary Material 5 [file 40942_2025_782_MOESM5_ESM.docx]

**Supplementary Material 5: Table 1**

**Article Title:**
Use of Retinal Ischemic Perivascular Lesions (RIPLs) as a Biomarker for Cardiovascular Disease – A Systematic Review and Meta-analysis

**Journal:**
International Journal of Retina and Vitreous

**Authors:**
Fatima Zahra, Manahil Malik, Khadijah Abid, Karim F. Damji, Haroon Tayyab

**Corresponding Author:**
Dr. Haroon Tayyab

**Affiliation:**
Department of Ophthalmology, Aga Khan University, Karachi, Pakistan

**E-mail Address:**
haroon.tayyab@aku.edu

Table 1: Baseline characteristics of included studies.

| **Study ID** | **Author** | **Year** | **Country** | **Study Design** | **RIPLs  definition** | **Exposure** | **Outcome** | **Sample Size** | **Average Age or Range** | **Gender (female %)** | **Race** |  |
| --- | --- | --- | --- | --- | --- | --- | --- | --- | --- | --- | --- | --- |
| Studies included in Meta-Analysis | | | | | | | | | | | | |
| 1 | Long et al.[15] | 2021 | USA | Cross-sectional, retrospective chart review | Presence of focal atrophy of the INL with a compensatory expansion of the ONL into the plane where the outer plexiform layer would be expected, leading to a wavy appearance of the middle retinal layers in OCT cross sections | Presence of RIPLs | CVD (Stroke, CHD) | 160 (CVD:84, control:76) | 68.8 | 61 | n=160 White=118(73.8)  Hispanic=12(7.5)  Asian=17(10.6)  African American = 1(0.6) |  |
| 2 | Drakopoulos et al. [14] | 2023 | USA | Cross-sectional | Presence of focal INL thinning with associated ONL expansion and displacement of the OPL without the presence of patient-reported scotoma | Presence of RIPLs | CVD | 36  (CAS:22, control:14) | CAS:48-84  Control: 55-80 | CAS=59  Control=64 | NA |  |
| 3 | Bakhoum et al.[16] | 2023 | USA | Retrospective cross-sectional | Presence of focal atrophy or thinning of the INL with a compensatory expansion of the ONL | Presence of RIPLs | Atrial fibrillation  (AF) | 197 (AF:106, control:91) | AF: 72.5 ± 7.8; Control: 70.6 ± 7.4 | AF: 47.2; Control: 48.4 | Control group(n=91)  White=64(70.3)  Asian=15(16.5)  Black=1(1.1) Multiracial=6(6.6) Others^†=^5(5.5)  AF group(n=106)  White=78(73.6)  Asian=11(10.4)  Black=3(2.8) Multiracial=12(11.3) Others^†^=2(1.9) |  |
| 4 | Bousquet et al.[13] | 2024 | USA, France, Egypt, Israel, Mexico | Retrospective cross-sectional | Presence of focal atrophy or thinning of the INL with expanded hypo reflectivity of the ONL on SD-OCT scan. | Presence of RIPLs | Myocardial infarction (MI) | 317  Total: 710 | MI: 65.9 ± 8.9; No MI: 68.1 ± 7.1 | MI: 27.8; No MI: 28.5 | NA |  |
| Remaining included Studies | | | | | | | | | | | | |
| 5 | Madala et al.[20] | 2022 | USA | case series | SD-OCT presence of focal atrophy of the INL associated with secondary expansion of the ONL/Henle’s fiber layer, leading to a wavy appearance of the middle retinal layers | Presence of RIPLs | CVD* | 11 | 44-80 | 54.5 | NA |  |
| 6 | Yeo et al.[21] |  | UK | case series | Focal atrophy of the INL alongside expansion of the ONL on SD-OCT | Presence of RIPLs | CVD | 11 | 48-94 | 18.0 | n=11 Asian=4(36.4) Caucasian=654.5) Arab=1(9.1) |  |

AF = atrial fibrillation

CAS = coronary artery syndrome

CVD= cardiovascular disease

CHD= coronary heart disease

INL= Inner Nuclear Layer

ONL= Outer Nuclear Layer

RIPLs= Retinal Ischemic Perivascular Lesions

* including multi-vessel CAD, significant carotid artery stenosis, soft carotid plaque, reduced cardiac ejection fraction, patent foramen ovale, cerebral infarction, subclavian steal syndrome and undiagnosed or poorly-controlled hypertension

^†^ Native Hawaiian or other Pacific Islander or unknown race.
