# Supplementary material for: Use of retinal ischemic perivascular lesions (RIPLS) as a biomarker for cardiovascular disease – a systematic review and meta-analysis
Source: Int J Retina Vitreous. 2025 Dec 24;12:15. doi: 10.1186/s40942-025-00782-2 (PMC12837118; doi:10.1186/s40942-025-00782-2)
Supplement: Supplementary file 6 — Supplementary Material 6 [file 40942_2025_782_MOESM6_ESM.docx]

**Supplementary Material 6: Table 2**

**Article Title:**
Use of Retinal Ischemic Perivascular Lesions (RIPLs) as a Biomarker for Cardiovascular Disease – A Systematic Review and Meta-analysis

**Journal:**
International Journal of Retina and Vitreous

**Authors:**
Fatima Zahra, Manahil Malik, Khadijah Abid, Karim F. Damji, Haroon Tayyab

**Corresponding Author:**
Dr. Haroon Tayyab

**Affiliation:**
Department of Ophthalmology, Aga Khan University, Karachi, Pakistan

**E-mail Address:**
haroon.tayyab@aku.edu

Table 2: Quality Assessment Table New Ottawa Scale

| Study | Year | Selection | Comparability | Exposure/Outcome | Total | Interpretation |
| --- | --- | --- | --- | --- | --- | --- |
| Long et al.[15] | 2021 | *** | ** | ** | 7 | Moderate-high quality |
| Drakopoulos et al. [14] | 2023 | **** | ** | ** | 8 | High quality |
| Bakhoum et al.[16] | 2023 | **** | ** | ** | 8 | High quality |
| Bousquet et al.[13] | 2024 | **** | ** | ** | 8 | High quality |
